# An Anillin-Ect2 Complex Stabilizes Central Spindle Microtubules at the Cortex during Cytokinesis

**DOI:** 10.1371/journal.pone.0034888

**Published:** 2012-04-13

**Authors:** Paul Frenette, Eric Haines, Michael Loloyan, Mena Kinal, Paknoosh Pakarian, Alisa Piekny

**Affiliations:** Department of Biology, Concordia University, Montreal, Quebec, Canada; Institut de Génétique et Développement de Rennes, France

## Abstract

Cytokinesis occurs due to the RhoA-dependent ingression of an actomyosin ring. During anaphase, the Rho GEF (guanine nucleotide exchange factor) Ect2 is recruited to the central spindle via its interaction with MgcRacGAP/Cyk-4, and activates RhoA in the central plane of the cell. Ect2 also localizes to the cortex, where it has access to RhoA. The N-terminus of Ect2 binds to Cyk-4, and the C-terminus contains conserved DH (Dbl homologous) and PH (Pleckstrin Homology) domains with GEF activity. The PH domain is required for Ect2's cortical localization, but its molecular function is not known. In cultured human cells, we found that the PH domain interacts with anillin, a contractile ring protein that scaffolds actin and myosin and interacts with RhoA. The anillin-Ect2 interaction may require Ect2's association with lipids, since a novel mutation in the PH domain, which disrupts phospholipid association, weakens their interaction. An anillin-RacGAP50C (homologue of Cyk-4) complex was previously described in *Drosophila*, which may crosslink the central spindle to the cortex to stabilize the position of the contractile ring. Our data supports an analogous function for the anillin-Ect2 complex in human cells and one hypothesis is that this complex has functionally replaced the *Drosophila* anillin-RacGAP50C complex. Complexes between central spindle proteins and cortical proteins could regulate the position of the contractile ring by stabilizing microtubule-cortical interactions at the division plane to ensure the generation of active RhoA in a discrete zone.

## Introduction

Cytokinesis describes the division of a cell into two genetically identical daughter cells and occurs due to the RhoA-mediated ingression of an actomyosin ring. The mitotic spindle determines the division plane during anaphase and is comprised of astral microtubules that emanate to the poles of the cell, and central spindle microtubules (including centrally positioned astral microtubules) that reach the equatorial cortex. While central spindle microtubules stimulate contractile ring formation in the center of the cell, astral microtubules inhibit the localization of contractile ring components at the poles of the cell [1], [2], [3]. The molecular components of the astral pathway have not been identified in many cell types, yet they may dominantly determine the division plane in large cells where the central spindle is positioned far from the cortex. Recent evidence also supports spindle-independent means of establishing the division plane [4], [5], which may occur via achieving a balance of polar vs. equatorial myosin activity [6].

The central spindle stimulates contractile ring formation by leading to the activation of RhoA by the GEF, Ect2 [7]. The centralspindlin complex, a heterotetramer of MKLP1 (kinesin-like protein) and MgcRacGAP/Cyk-4 [8], [9], helps form the central spindle in anaphase. Ect2 binds to Cyk-4 via N-terminal BRCT (BRCA1 C-terminus) domains, which recruits Ect2 to the central spindle [10], [11], [12]. Ect2's GEF activity is mediated by conserved DH and PH domains in its C-terminus [13]. The DH domain catalyzes nucleotide exchange on RhoA and the PH domain contributes to Ect2's cortical localization, although its molecular function is not known (e.g. phospholipid or protein interactions) [13], [14], [15], [16]. In metaphase, Cdk1 phosphorylation causes a conformational change in Ect2, which blocks Cyk-4 binding and inhibits its GEF activity [11], [15]. Formation of the Cyk-4-Ect2 complex also requires Cyk-4 phosphorylation by Plk1 (Polo kinase 1) [17], [18], [19], [20], [21]. Human cells treated with a Plk1 inhibitor, Cyk-4 RNAi or Ect2 RNAi have decreased RhoA localization and fail to form a contractile ring, suggesting that Ect2 must bind to Cyk-4 to generate active RhoA and initiate cytokinesis [11], [12], [20], [21], [22]. Coupling Ect2 activation to central spindle assembly ensures that RhoA is activated only after sister chromatids have segregated.

The mechanism that maintains a well-positioned contractile ring is not fully understood. For example, although we understand how Ect2 is recruited to the central spindle, it is not clear how active RhoA stays localized in a discrete zone rather than spreading throughout the cortex. One hypothesis is that a GAP (GTPase activating protein; possibly Cyk-4) down regulates RhoA at the same time that Ect2 activates RhoA. Consistent with this model, in *Xenopus* and echinoderm cells, disruption of Cyk-4's GAP activity causes expansion of the active RhoA zone [23]. However, in human cells, Cyk-4 depletion blocks RhoA localization (and likely its activation) [11] and in *C. elegans*, CYK-4's GAP activity may regulate other GTPases [24]. Since RhoA is at the cortex, and in yeast is preferentially associated with phospholipids such as PI_4,5_P_2_ [25], one hypothesis is that Ect2's activity (ability to exchange nucleotide or access to substrate) is influenced by its cortical localization and controlling this localization in the equatorial plane could generate a discrete zone of active RhoA. Although Ect2 is enriched on the central spindle, it also localizes cortically in human cells and is primarily cortical in *Drosophila* cells [14], [26] where RacGAP50C (Cyk-4)/Pebble (Ect2) complexes localize as a ring that overlaps with the cortex [10]. Furthermore, the C-terminal PH region was shown to mediate Ect2's cortical localization [14], [26].

Anillin is a key component of the ring and through its many interactions (*i.e.* with actin, myosin and septins), functions as a scaffold to stabilize the division plane during cytokinesis [27]. In human and *Drosophila* cells, anillin depletion causes cortical oscillations due to lateral instability of the contractile ring, followed by furrow regression [28], [29], [30], [31], [32]. Human anillin also interacts with RhoA and its C-terminal anillin homology domain (AHD) shares homology with the RhoA-GTP binding protein Rhotekin [30]. Furthermore, RhoA is not stabilized by fixation in anillin-depleted cells, supporting it may feed back to influence the localization of active RhoA [30], [32]. In addition, anillin may tether central spindle microtubules, as *Drosophila* anillin was shown to interact with RacGAP50C, a homologue of Cyk-4 [33], [34]. An attractive hypothesis is that anillin's interaction with RacGAP50C may stabilize the division plane by anchoring central spindle microtubules at the equatorial cortex to control the generation of active RhoA.

Identifying the mechanism that maintains the division plane is central to our understanding of cytokinesis. Ect2 activates RhoA for contractile ring formation and ingression, yet little is known regarding Ect2's molecular interactions at the cortex. The PH domain is required for Ect2's cortical localization and we found an interaction between the PH domain of Ect2 and the AHD of anillin. This interaction may require Ect2's association with phospholipids, since a novel mutation in the PH domain of Ect2 disrupts its association with phospholipids and weakens the anillin-Ect2 interaction. Our results suggest that their interaction may physically link central spindle microtubules with the contractile ring, a function that was previously described for the anillin-RacGAP50C complex in *Drosophila*. We found that the *Drosophila* Ect2 homologue, Pebble (Pbl), does not interact with anillin and one hypothesis is that the anillin-Ect2 interaction in human cells is functionally analogous to the anillin-RacGAP50C interaction in *Drosophila*. This linkage between the cortex and the central spindle microtubules could promote the stable activation of RhoA in a discrete zone.

## Results

### Anillin interacts with an Ect2 complex during cytokinesis

**Figure 1:**
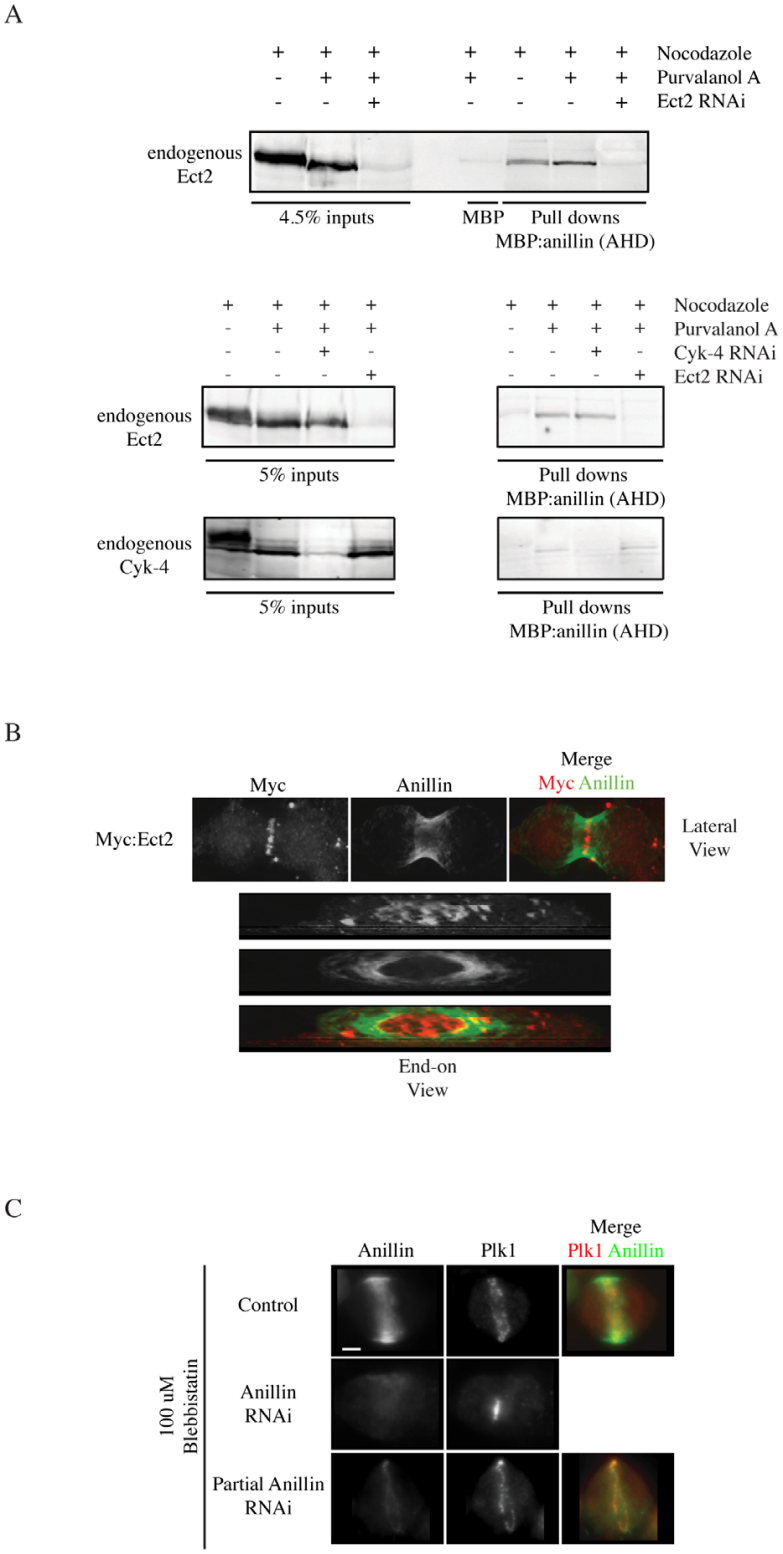
Anillin interacts with Ect2 during cytokinesis. A) Western blots show the interaction of endogenous Ect2 (and Cyk-4) with the AHD of anillin. The top western blot shows Hela lysates from cells treated with nocodazole, purvalanol and/or Ect2 RNAi pulled down with MBP or MBP:AHD and probed for endogenous Ect2. The bottom western blot shows Hela lysates from cells treated with nocodazole, purvalanol and/or Cyk-4 RNAi and/or Ect2 RNAi pulled down with MBP:AHD and probed for endogenous Ect2 (top panel) or Cyk-4 (bottom panel). B) Z-stack projections of MeOH-fixed Hela cells transfected with Myc:Ect2 and co-stained for Myc (red) and anillin (green). Both a lateral view and an end-on view are shown. C) Z-stack projections of MeOH-fixed Hela cells+/−anillin RNAi treated with 100 µM Blebbistatin to inhibit oscillations and stained for Plk1 (red) and anillin (green). Scale bar is 10 µm.

**Figure 2:**
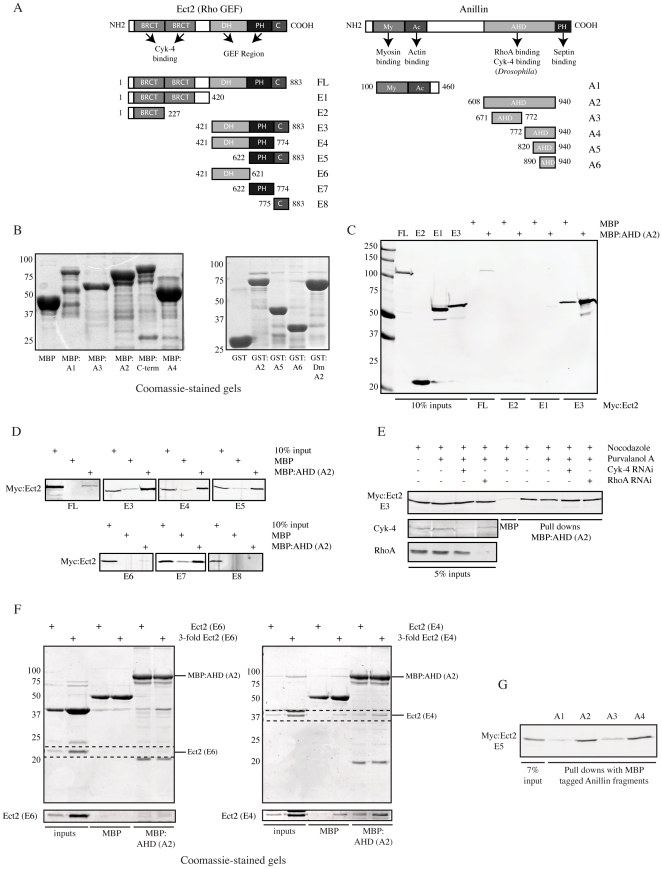
The PH region of Ect2 interacts with the AHD of anillin. A) Structures of Ect2 (BRCT: BRCA1 C terminus domain, DH: Dbl homology, PH: Pleckstrin Homology, C: C-region) and anillin (My: Myosin, Ac: Actin, AHD: Anillin Homology Domain, PH: Pleckstrin Homology). B) Coomassie-stained gels show the various MBP and GST-tagged recombinant anillin proteins used in this study. The MBP-tagged proteins are shown in the gel on the left, and the GST-tagged proteins are shown in the gel on the right. C) A western blot of lysates from HEK-293 cells transfected with Myc: Ect2 FL, E1 (N-term), E2 (BRCT) and E3 (C-term) pulled down with MBP or MBP:AHD (A2) of anillin and stained for Myc. D) Western blots of lysates from HEK-293 cells transfected with Myc-tagged Ect2 constructs (FL, E3–E8) pulled down with MBP or MBP:AHD of anillin stained for Myc. E) Western blots of lysates from Hela cells expressing Myc:Ect2 (E3) treated with nocodazole, purvalanol A, and Cyk-4 or RhoA RNAi, and pulled down with MBP or MBP:AHD of anillin, stained for Myc, Cyk-4 and RhoA. F) Coomassie-stained gels show recombinant, cleaved Ect2 fragments [E6 (DH domain) or E4 (DH+PH domains)] pulled down with MBP or MBP:AHD. Boxes outline E6 or E4, and are shown below. G) A western blot of lysates from HEK-293 cells transfected with Myc:Ect2 (E5) pulled down with anillin fragments (A1, A2, A3 and A4) tagged with MBP and stained for Myc.

Ect2 localizes to the central spindle and to the cortex during cytokinesis. PH and C domains in the C-terminus of Ect2 mediate its cortical localization. However, little is known regarding the molecular function of these domains. Previous studies showed that *Drosophila* anillin, a cortical protein, interacts with RacGAP50C (Cyk-4 homologue) *in vitro* and *in vivo*, which may crosslink the central spindle to the overlying cortex [33], [34]. We determined if human anillin interacts with Ect2 and Cyk-4 during cytokinesis. Lysates from synchronized Hela cells were pulled down with 200 nM recombinant anillin AHD (Figure 1A; recombinant protein shown in Figure 2B). Ect2 was pulled down from cells that had been treated with both nocodazole (disrupts microtubule polymerization and arrests cells in metaphase) and purvalanol A (inhibits Cdk1 activity to promote mitotic exit) [21], but only weakly from cells treated with nocodazole alone (Figure 1A). To determine if Cyk-4 also interacts with anillin, a similar experiment was performed and blots were probed for both Ect2 and Cyk-4. Low amounts of Cyk-4 were simultaneously pulled down with Ect2 from purvalanol-treated cells (Figure 1A). The AHD from *Drosophila* anillin directly binds to RacGAP50C *in vitro* [33], yet Hela cells depleted of endogenous Cyk-4 had no effect on anillin's interaction with Ect2 (Figure 1A). Similarly, anillin interacted with Cyk-4 after Ect2-depletion (Figure 1A). Therefore, independent anillin-Ect2 and anillin-Cyk-4 complexes may form, or a common complex may form that is mediated by a different protein and/or lipids.

We also examined the colocalization of Ect2 and anillin during cytokinesis. Z-stack projections of methanol-fixed Hela cells expressing Myc:Ect2 were co-stained for Myc and anillin (Figure 1B). The stacks were rotated to show an end-on view of the contractile ring (Figure 1B lower panels). Although it is difficult to see colocalization from the lateral view of the cell, the end-on view shows colocalization between the outermost pools of Ect2 and innermost pools of anillin during furrow ingression (Figure 1B). Anillin localizes strongly to the cortex and we partially depleted anillin to observe localization of the remaining protein (cells were treated with blebbistatin to inhibit the oscillatory phenotype; Figure 1C). The remaining pools of anillin localized to a discrete band that overlapped with Plk1, a central spindle protein (Figure 1C). The recruitment of Ect2 and anillin to similar cellular locations, including the outermost cortically associated central spindle microtubules, supports them being in a common complex *in vivo*.

### The PH domain of Ect2 interacts with the AHD of anillin

Anillin interacted with Ect2 and Cyk-4, but its interaction with Ect2 appeared to be more robust vs. Cyk-4 (Figure 1A) and we determined the minimal binding regions on both Ect2 and anillin. Schematics showing the structures of Ect2 and anillin are shown in Figure 2A and the recombinant anillin proteins that were used in this study are shown in Figure 2B. To identify the interacting region on Ect2, Myc-tagged proteins containing various Ect2 fragments were pulled down from HEK-293 cell lysates with 200 nM of recombinant MBP:anillin (A2; vs. 700 nM MBP control protein; Figure 2C and Figure S1A). The AHD of anillin associated more strongly with the C-terminus (E3) vs. full-length Ect2, and did not interact with the N-terminus (E1 or E2; Figure 2C and Figure S1A). Since Cyk-4 interacts with the N-terminus of Ect2, this result supports other data showing that anillin's interaction with Ect2 is independent of Cyk-4 (Figure 1A). HEK-293 lysates from cells transfected with Myc-tagged constructs containing various C-terminal fragments of Ect2 were pulled down with 200 nM recombinant MBP:anillin (A2; Figure 2D). Any piece containing the PH domain (E3, E4, E5 and E7) interacted with anillin, but the DH (E6) and C (E8) regions alone did not (Figure 2D). These results suggest that the PH domain of Ect2 is the minimal interacting region with anillin.

**Figure 3:**
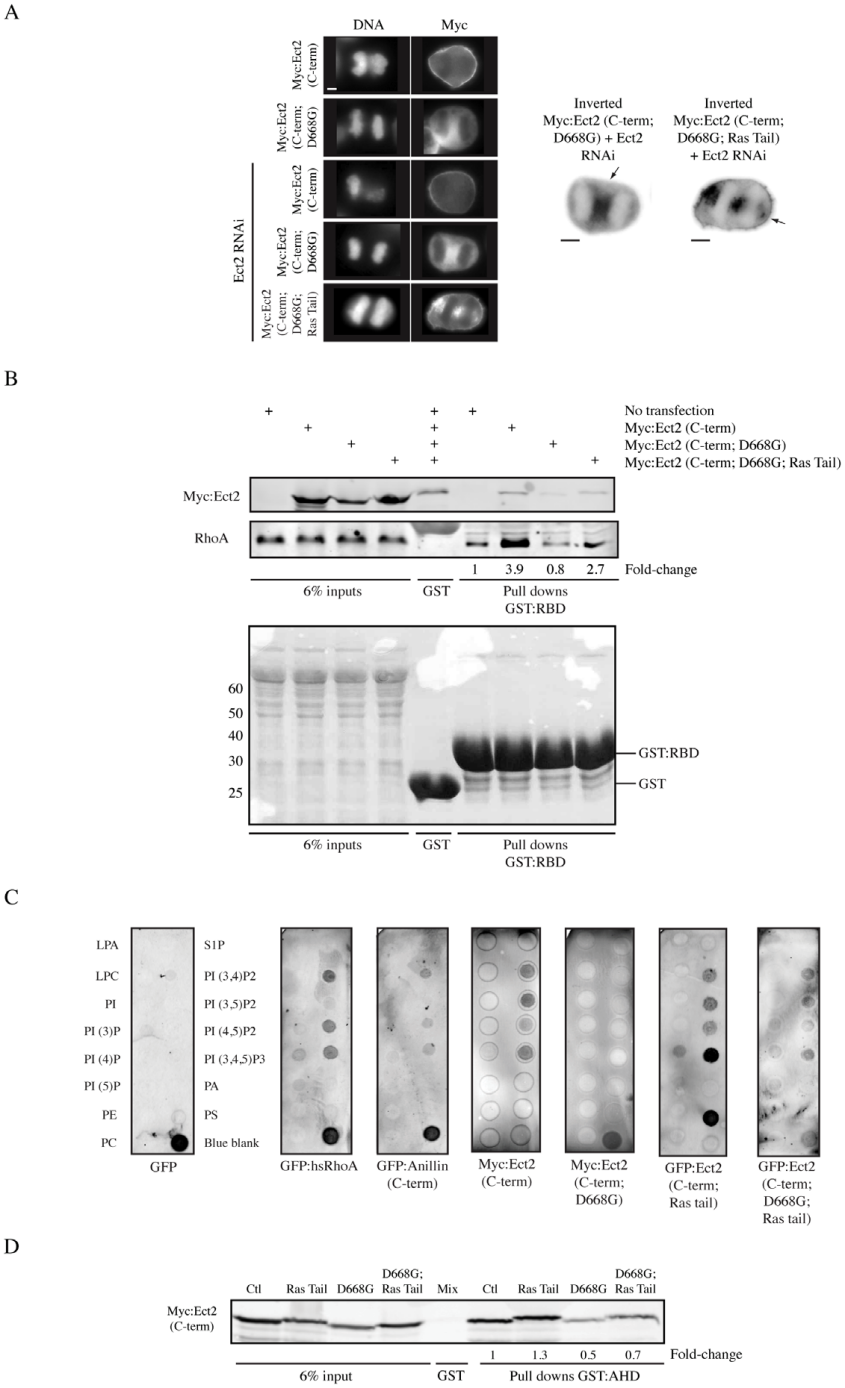
Anillin interacts with an Ect2 complex at the cortex. A) Single plane images of fixed Hela cells transfected with Myc:Ect2 C-terminal constructs+/−Ect2 siRNAs co-stained for DNA (DAPI) and Myc. Inverted images show Myc localization for D668G and D668G; Ras Tail. Arrows point to the cortex. Scale bar is 10 µm. B) A western blot of active RhoA pulled down with GST:RBD using lysates from HEK-293 cells expressing Myc:Ect2 C-terminal constructs. The fold-change of RhoA binding vs. control is indicated for each lane. A ponceau stain of the blot is shown below. C) Western blots of PIP strips incubated with lysates from HEK-293 cells expressing GFP, GFP:RhoA, GFP:anillin (C-term), Myc:Ect2 (C-term wt or D668G), and GFP:Ect2 (C-term; Ras Tail or D668G; Ras Tail). D) A western blot of lysates from HEK-293 cells transfected with Myc:Ect2 (E3 wt, Ras Tail, D668G or D668G; Ras Tail) pulled down with GST:AHD of anillin and stained for Myc.

The PH domain is in the C-terminus of Ect2, which is auto inhibited in metaphase, and could explain why anillin and Ect2 interact more strongly after Cdk1 inhibition (relieves the auto inhibition of Ect2 by permitting binding to Cyk-4) [11], [15]. Therefore, the C-terminus of Ect2 should be able to interact with anillin independent of cell cycle stage after removing the N-terminus. Indeed, 200 nM MBP:anillin (A2) interacted strongly with E3 regardless of the state of Cdk1 activity (nocodazole vs. nocodazole and the Cdk inhibitor purvalanol A; vs. 700 nM MBP; Figure 2E). E3 localizes to the cortex of mitotic cells (Figure 3A) and the stronger interaction between anillin and E3 vs. FL suggests that they may interact near/at the cortex. Indeed, anillin co-localized with E3 in cortical filamentous structures in anaphase cells over-expressing E3 (Figure S1B).

Since the AHD of anillin and the C-terminus of Ect2 both interact with RhoA, we determined if the anillin-Ect2 interaction is RhoA-dependent. The C-terminus of Ect2 was pulled down by MBP:anillin (A2) after depletion of endogenous RhoA by RNAi (Figure 2E). Furthermore, mutations in the C-terminus of anillin (837 DFEINIE 843 to AFAINIA) that decrease the anillin-RhoA interaction [30] had no effect on the interaction between anillin and E3 (200 nM each; Figure S1C). Thus, the interaction between the AHD of anillin and E3 occur independently of RhoA and could be direct. A weak interaction was detected between recombinant MBP:anillin (A2) and a C-terminal fragment of Ect2 that contains the PH domain *in vitro* (E4 vs. the DH domain alone (E6); Figure 2F). While this result supports a direct interaction between anillin and Ect2, their weak association suggests that other proteins or lipids may mediate their interaction *in vivo*. Anillin shows specificity in its interaction with Ect2, since 200 nM of recombinant GST:anillin (A2) pulled down E3 more strongly than a PH domain fragment from MyoGEF (a RhoGEF that also functions in cytokinesis and contains a DH PH domain motif [35], [36]; Figure S1D).

The minimal Ect2 interaction domain on anillin also was determined. Bacterially expressed GST or MBP-fused proteins containing various regions of anillin were used to pull down Myc-tagged Ect2 (E3 and E5) fragments from HEK-293 cell lysates. The C-terminal part of the AHD (A6; 200 nM) pulled down Ect2 similar to larger AHD fragments (A2, A4 and A5 vs. A1; 200 nM each; Figure 2G and Figure S1E). However, the N-terminal part of the AHD (A3) only weakly interacted with Ect2 (200 nM; Figure 2G). These data indicate that the primary determinant of Ect2 binding lies in the C-terminus of the AHD.

### Anillin's interaction with the Ect2 complex requires Ect2's association with phospholipids

We uncovered an interaction between the PH domain of Ect2 and the AHD of anillin. The PH domain mediates the cortical localization of Ect2 [14], [26], and we determined if this localization is required for its interaction with anillin. We generated point mutations in the PH domain to disrupt its function. Ect2 is conserved across metazoans and a sequence alignment (Clustal W) of the PH domain from Human Ect2, *Drosophila* Pebble and *C. elegans* ECT-2 was performed to look for clusters of highly conserved residues (Figure S2A). Mutations (seven sets, see Materials and Methods; data not shown) were generated in the C-terminus of Ect2, which localizes cortically (Figure 3A) and activates RhoA (determined by pull down experiments using 1 µM of GST tagged with Rho-GTP binding domain (RBD) from Rhotekin [37]; Figure 3B). One mutation, a conserved Aspartate (D) at position 668 to Glycine (G), abolished Ect2's cortical localization (Figure 3A), decreased Ect2's generation of active RhoA (0.8 fold+/−0.4 standard deviation (S.D.) change in active RhoA levels compared to 3.9+/−1.4 S.D. for wild-type (wt); Figure 3B), and disrupted its interaction with phospholipids (Figure 3C). To determine the effect of the PH domain mutation on anillin binding, pull downs were performed using the AHD from anillin and the Ect2 (C-term; D668G) mutant from cell lysates. As shown in Figure 3D, the mutant protein was not pulled down as effectively as wild-type Ect2 (0.5-fold lower; comparisons were normalized based on inputs). However, it is possible that the affinity between Ect2 (C-term) and the AHD is quite low, and Ect2 binding could decrease proportionately at lower concentrations. To better assess the effect of the D668G mutation on the Ect2-anillin interaction, lysates from HEK-293 cells transfected with Ect2 (C-term) or Ect2 (C-term; D668G) were diluted by 0.5-fold, 0.25-fold or 0.05-fold, and pulled down with 200 nM GST-tagged AHD (Figure S2B). At all dilutions, more wt Ect2 bound to the AHD vs. mutant Ect2. Furthermore, wt Ect2 (C-term) showed high affinity for the AHD, with high amounts of protein binding (38%) at the lowest dilution. This data supports that Ect2's cortical localization and lipid association may strengthen the anillin-Ect2 interaction.

We determined if re-localizing the mutant protein back to the membrane is sufficient to restore anillin binding. The CAAX box of k-Ras (Ras tail; EKMSKDGKKKKKKSKTKCVIM) was added to Ect2 (C-term; D668G). Adding the Ras tail onto wt Ect2 (C-term) enhanced its phospholipid-binding profile, as it allowed interaction with one additional phospholipid (Figure 3C). Adding the Ras tail to mutant Ect2 (C-term) partially restored cortical localization (Figure 3A), partially restored the generation of active RhoA (2.7+/−1.5 S.D. fold change vs. 3.9+/−1.4 S.D. for wt and 0.8+/−0.4 S.D. for D668G; Figure 3B), and restored the phospholipid-binding profile (Figure 3C). We tested if re-localizing the mutant protein back to the membrane could restore anillin binding. Pull downs were performed using 200 nM of bacterially expressed and purified GST-tagged AHD and lysates from HEK-293 cells expressing Myc-tagged Ras-tail modified Ect2 (C-term) mutant (Figure 3D). Adding the Ras tail to the mutant conferred increased anillin binding, but not to the same level as wild-type Ect2 (C-term). Interestingly, adding the Ras tail to wt Ect2 (C-term) also increased its interaction with anillin (Figure 3D). These results support that anillin preferentially interacts with cortically localized/phospholipid-associated Ect2.

### Anillin's interaction with an Ect2 complex may be required for the cortical localization of central spindle proteins

**Figure 4:**
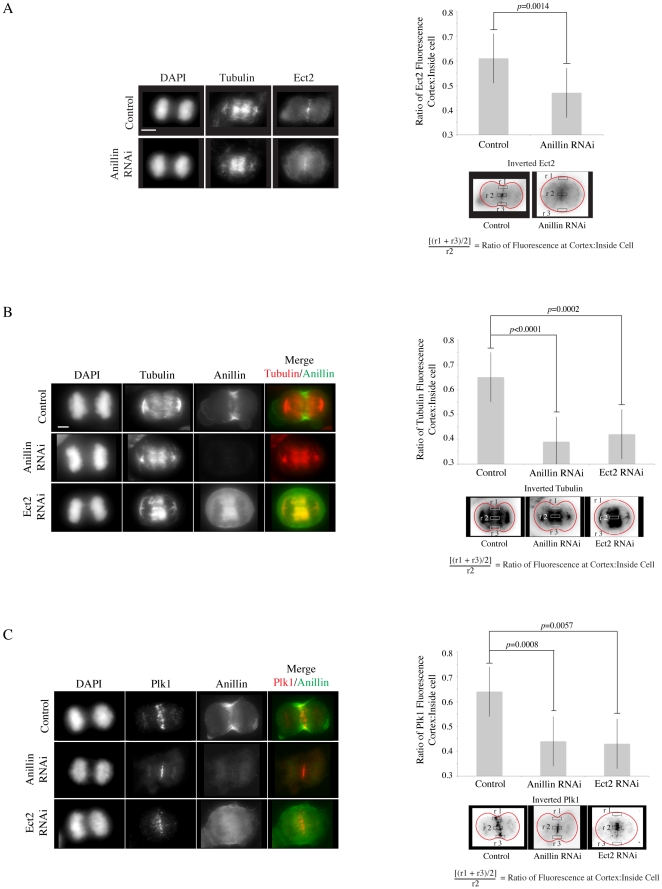
A complex that includes anillin and Ect2 may stabilize the cortical localization of central spindle microtubules. A) Z-stack projections of fixed Hela cells transfected with anillin siRNA co-stained for tubulin, Ect2 and DAPI. The graph shows the average ratios of Ect2 fluorescence near the equatorial cortex vs. inside the cell for control and anillin-depleted cells. Bars on all graphs show standard deviation and probabilities are from students t tests. B) Z-stack projections of fixed Hela cells transfected with anillin or Ect2 siRNA co-stained for DAPI, tubulin (red) and anillin (green). The graph shows average ratios of tubulin fluorescence near the equatorial cortex vs. inside the cell for control, anillin and Ect2-depleted cells. C) Z-stack projections of fixed Hela cells transfected with anillin or Ect2 siRNA co-stained for DAPI, Plk1 (red) and anillin (green). The graph shows average ratios of Plk1 fluorescence near the equatorial cortex vs. inside the cell for control, anillin and Ect2-depleted cells. Scale bar is 10 µm.

Our data supports an interaction between Ect2 and anillin at the cortex. In human and *Drosophila* cells, anillin depletion causes lateral instability of the contractile ring and loss of TCA-fixed RhoA suggesting it stabilizes the division plane and feeds back to upstream pathways [28], [29], [30], [31], [32]. In *Drosophila*, anillin interacts with RacGAP50C (Cyk-4 homologue) [33], [34], possibly to crosslink the central spindle with the cortex and we hypothesize that the anillin-Ect2 interaction may have a similar function in human cells. First, the average ratio of Ect2 fluorescence near the equatorial cortex vs. inside the cell (central spindle region) was measured and graphed for control cells (n = 11) vs. anillin-depleted cells (n = 13; Figure 4A). Pools of Ect2 that localize near the cortex significantly decreased in anillin RNAi cells in comparison to control cells. Anillin-depleted cells also had lower levels of Ect2 at the central spindle, as shown by line plots measuring Ect2 fluorescence across the equatorial plane (n = 9 control cells vs. n = 11 anillin RNAi cells; Figure S3A). To determine if the change in Ect2 localization reflects a loss in central spindle microtubules near the cortex, the localization of tubulin and Plk1 (a central spindle protein) was examined in anillin-depleted cells. The localization of tubulin and Plk1 near the cortex significantly decreased in comparison to control cells (for tubulin, n = 11 control cells and 10 anillin RNAi cells and for Plk1, n = 12 control cells and 13 anillin RNAi cells; Figure 4B, C and Figure S3B; also co-stained for nonmuscle myosin II and tubulin, n = 15 control cells and 18 anillin RNAi cells; Figure S3C). If the anillin-Ect2 interaction is required to stabilize microtubules near the cortex, then Ect2 depletion should cause a phenotype similar to anillin RNAi. Indeed, in Ect2-depleted cells, the localization of microtubules and Plk1 near the cortex decreased in comparison to control cells (for tubulin, n = 10 Ect2 RNAi cells and for Plk1, n = 11 Ect2 RNAi cells; Figure 4B, C; also co-stained for nonmuscle myosin II and tubulin, n = 13 Ect2 RNAi cells; Figure S3C). This data suggests that Ect2 and anillin may crosslink centrally-positioned microtubules at the equatorial cortex, similar to the anillin-RacGAP50C complex in *Drosophila*.

### *Drosophila* Pebble (Ect2 homologue) does not interact with anillin

**Figure 5:**
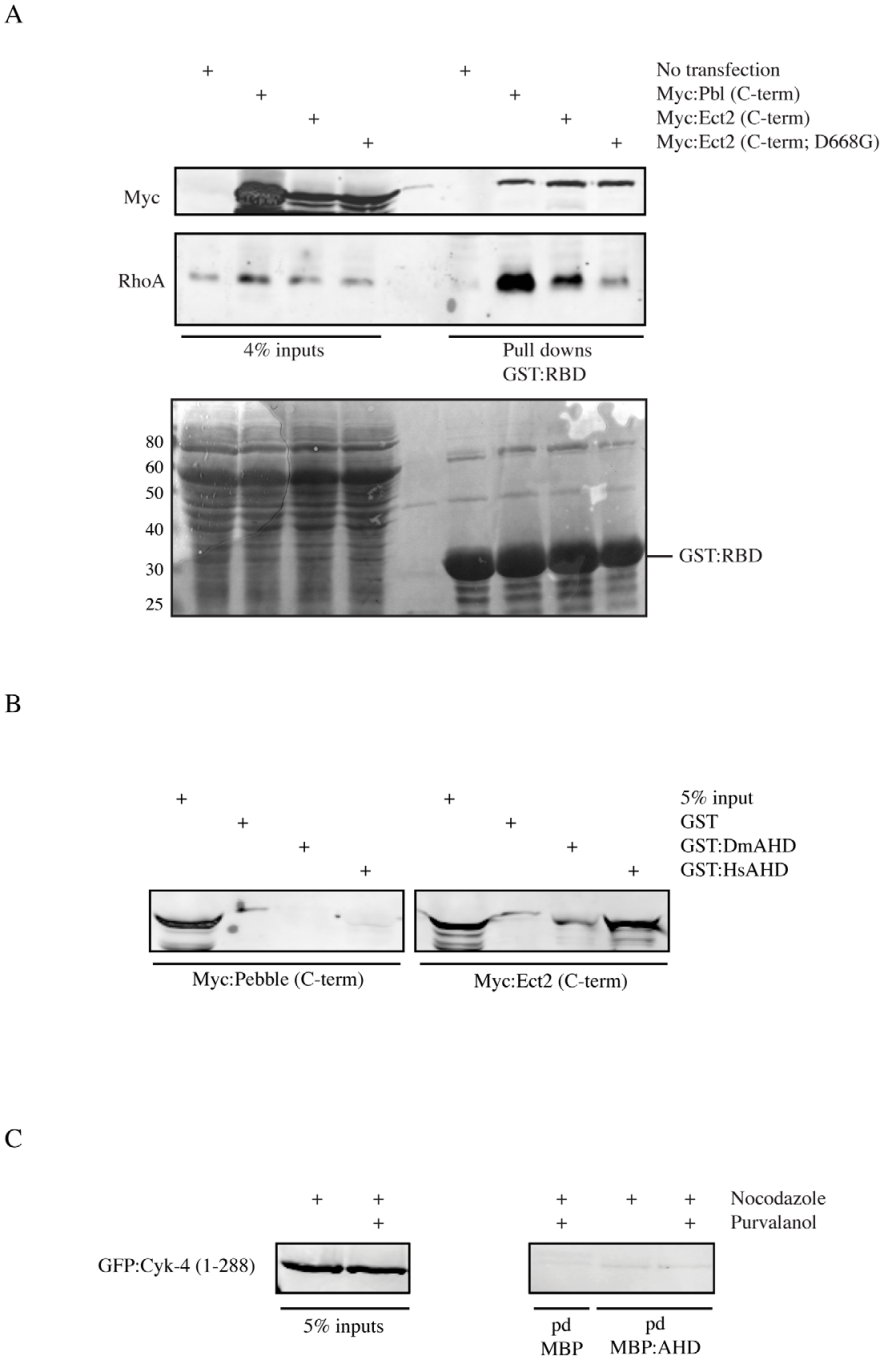
*Drosophila* Pbl does not interact with the AHD of *Drosophila* and human anillin. A) A western blot shows the levels of active RhoA pulled down with GST:RBD using lysates from HEK-293 cells transfected with Pbl or Ect2 C-term constructs. The blot was probed for RhoA and Myc as indicated, and a ponceau stain of the blot is shown below. B) A western blot of lysates from HEK-293 cells transfected with Myc-tagged Pbl or Ect2 C-term constructs pulled down with GST-tagged AHD from *Drosophila* (Dm) or human (Hs) anillin probed for Myc. C) A western blot of lysates from Hela cells transfected with GFP-tagged Cyk-4 (1–288), pulled down with MBP-tagged AHD of human anillin and probed for GFP.

*Drosophila* anillin forms a complex with RacGAP50C *in vivo* and *in vitro* and Pebble (Ect2 homologue) was not identified as a component of this complex [33], [34]. *Drosophila* Pebble interacts with RacGAP50C and localizes primarily to the cortex [10] vs. human Ect2, which localizes to the cortex and central spindle microtubules [11]. We determined if the anillin-Ect2 interaction is conserved in other organisms and, particularly, if *Drosophila* anillin interacts with Pebble. First, we examined the activity of the C-terminus of Pebble (Pbl) in HEK-293 cells. Lysates from HEK-293 cells transfected with Myc-tagged Ect2 (C-term) or Pbl (C-term) contained higher levels of RhoA-GTP (based on RBD pull down assays) compared to non-transfected cells or cells transfected with Myc-tagged Ect2 (C-term; D668G) (Figure 5A). Next, pull down assays were performed to assess anillin's ability to interact with Pbl (C-term). Bacterially expressed and purified GST-tagged AHD of human (Hs) or *Drosophila* (Dm) anillin (200 nM) was used to pull down Ect2 (C-term) and Pbl (C-term) from HEK-293 lysates. Although *Drosophila* AHD weakly interacted with human Ect2, neither human nor *Drosophila* AHD interacted with Pbl (Figure 5B and Figure S4). Therefore, an evolutionary change may have occurred in Pebble or human Ect2 that altered their ability to interact with anillin. As shown in Figure 1A, a small amount of endogenous Cyk-4 was pulled down by the AHD of human anillin. Since fragments of Ect2 bind to anillin more robustly vs. full-length, we determined if anillin interacts more strongly with the N-terminus of Cyk-4 (1–288; corresponds to the region from RacGAP50C predicted to interact with *Drosophila* anillin). However, Cyk-4 (N-term) did not interact with the AHD from human anillin (Figure 5C). This suggests that a change also may have occurred in anillin and/or Cyk-4 to alter their affinity in human cells. We hypothesize that the human anillin-Ect2 interaction is functionally analogous to the *Drosophila* anillin-RacGAP50C interaction, which crosslinks the mitotic spindle to the cortex, to stabilize the position of the division plane during cytokinesis.

## Discussion

Ect2 generates active RhoA for successful cytokinesis in metazoans. Although we understand the mechanism that recruits Ect2 to the central spindle, it is not clear how a discrete zone of active RhoA is maintained in the overlying cortex. Ect2 localizes to the cortex via its PH domain [14], [26], but the molecular function of this domain is not known. Here we describe an interaction between the PH domain of Ect2 and the AHD of anillin. *Drosophila* anillin interacts with RacGAP50C (Cyk-4 homologue) *in vivo* and *in vitro*, which may stabilize microtubules at the equatorial cortex [33], [34]. Our results support that the anillin-Ect2 interaction could have a similar function. The *Drosophila* Ect2 homologue, Pebble, does not interact with anillin and we propose that an evolutionary change has favored the formation of an anillin-RacGAP50C complex in *Drosophila* and/or an anillin-Ect2 complex in human cells. In metazoans, the formation of stable cortical-spindle interactions at the equatorial cortex may ensure the generation of a discrete zone of active RhoA to maintain the division plane.

We found that the AHD of human anillin interacts with Ect2 and Cyk-4, and these interactions preferentially occurred in lysates from Cdk1-inhibited cells (to stimulate mitotic exit). Anillin interacted simultaneously with Ect2 and Cyk-4, although its interaction with Ect2 was more robust vs. Cyk-4. Furthermore, their interaction with anillin was independent of each other. This suggests that anillin interacts with a protein that is common to the Ect2/Cyk-4 complex, or that anillin can associate with multiple complexes (containing Ect2 or Cyk-4), and may have a higher affinity for the Ect2 complex. Since the interaction between anillin and Ect2 or Cyk-4 preferentially occurred after Cdk1 inhibition, this suggests that removal of Cdk1 phosphorylation(s) and/or phosphorylation(s) by anaphase-specific kinases increases their affinity for anillin. Dephosphorylation of a Cdk1 site on Ect2 (T341) causes Ect2 to undergo a conformational change that permits it to form a complex with Cyk-4 [11], [12]. This same conformational change may also support its interactions with other proteins or lipids. Ect2 has additional phosphorylation sites for cell cycle kinases, and these other sites could regulate Ect2's interaction with other proteins or lipids [16], [19].

The interaction between anillin and Ect2 may be RhoA independent. Removing endogenous RhoA via RNAi or using a mutant version of anillin that disrupts RhoA binding had no affect on anillin's interaction with the C-terminus of Ect2, suggesting that the binding sites for RhoA and Ect2 are separable. However, RhoA recruits anillin to the equatorial cortex during cytokinesis [29], [30], and complexes containing Ect2, RhoA and anillin likely are formed *in vivo*. It is not clear how these complexes could form and also permit anillin's interactions with filamentous proteins including myosin, actin and septins. Recently, it was shown that the two halves (N and C-terminal) of anillin are spatially and functionally separable [38]. The N-terminus of anillin interacts with actin and myosin and contributes to midbody formation, while the C-terminus interacts with RhoA and septins, and localizes to the membrane [38].

Ect2's GEF activity requires DH and PH domains in its C-terminus. As described above, the N-terminus of Ect2 binds to Cyk-4, which recruits Ect2 to the central spindle. Ect2 also localizes to the cortex via PH and C domains in its C-terminus [14], [39]. A recent study showed that Ect2's cortical localization is essential for its function *in vivo*, possibly by positioning Ect2 close to its substrate, RhoA [39]. Using a novel mutation in Ect2's PH domain, we found that this region mediates interactions with phospholipids, and is essential for Ect2's cortical localization and GEF activity *in vivo*. Ect2's GEF activity was not fully restored by targeting the mutant back to the membrane using the tail from k-Ras. Therefore, association with phospholipids may be required to generate active RhoA, however, there are unique properties associated with the PH domain that cannot be replaced by the Ras tail. The role of the PH domain in Ect2's ability to exchange nucleotide on RhoA still needs to be determined, based on conflicting results from several *in vitro* assays [13], [39]. Phospholipids are important regulators of cytokinesis, particularly PI_(4,5)_P_2_, which is enriched in the cleavage furrow and localizes RhoA [25], [40], [41], [42]. However, it is not clear if the main role of phospholipids is to provide Ect2 with better access to its substrate, or if they also potentiate Ect2's GEF activity.

The interaction between the PH domain of Ect2 and the AHD of anillin may require Ect2's association with phospholipids, suggesting that it occurs at the cortex vs. other locations in the cell. In support of this, colocalization was observed between anillin and Ect2 near the cortex and one hypothesis is that the anillin-Ect2 complex could crosslink mitotic spindle microtubules (via Ect2) to the cortex (via anillin).

*Drosophila* anillin and RacGAP50C (Cyk-4 homologue) directly interact, and may crosslink the central spindle to the overlying cortex to maintain the division plane [33], [34]. We hypothesize that the anillin-Ect2 interaction similarly stabilizes the division plane in human cells, since we observed a decrease in the proportion of cortically localized central spindle-labeled microtubules in anillin and Ect2-depleted cells in comparison to control cells. Physically crosslinking the central spindle to the cortex via Ect2 could maintain the generation of active RhoA in a discrete plane. In support of this, in anillin-depleted cells, TCA-fixed RhoA is lost and cells form unstable furrows that ingress partially, undergo oscillations and regress [30], [32]. We were unable to detect interactions between Pebble (*Drosophila* Ect2 homologue) and *Drosophila* or human anillin, and observed a weak interaction between human anillin and endogenous Cyk-4. Therefore, in human cells, an interaction between anillin and Ect2 could have evolved to functionally replace the *Drosophila* anillin-RacGAP50C complex, or vice versa. Part of the reason for this change could be due to Ect2's localization, which is at both the cortex and central spindle in human cells, but is primarily cortical in *Drosophila* cells. Since many filamentous proteins crowd the cortex, this could restrict the formation of complexes with mitotic spindle microtubules vs. proteins that are shifted into different spatial compartments.

A physical link between the central spindle and the cortex has been previously described, and it was proposed that signals associated with the central spindle communicate with the overlying cortex to form and ingress the contractile ring [10], [43], [44]. Although some of the signals that initiate contractile ring formation were determined [11], [17], [19], [20], [21], it was not known how active RhoA, the upstream regulator for the ring, is generated in a specific location at the cortex. A common theme for metazoans could be to physically crosslink the spindle to the cortex via an interaction between a central spindle protein (Ect2 or RacGAP50C) and a cortical protein (anillin). This could stabilize central spindle microtubules at the equatorial cortex to promote the generation of RhoA by Ect2.

## Materials and Methods

### Cell Culture and Transfection

HEK-293 and Hela cells (generously provided by Dr. Glotzer, The University of Chicago, Chicago, IL) [11] were maintained in DMEM high glucose media supplemented with 10% FBS, 2 mM L-glutamine, 100 u penicillin and 0.1 mg/mL streptomycin as previously described [11]. Cells plated in media without antibiotics were transfected with siRNAs using Oligofectamine (Invitrogen) or co-transfected with DNA using Lipofectamine 2000 (Invitrogen). For optimal results with Lipofectamine, we used ∼4-fold less reagent than recommended and transfected cells above 70% confluency.

### RNAi and Drug treatments

Anillin, Cyk-4, RhoA and Ect2 RNAi were performed as previously described [11], [30]. Cells were synchronized in metaphase using 40 ng/mL nocodazole (Sigma-Aldrich) and were stimulated to exit mitosis using 22.5 µM purvalanol A (Sigma-Aldrich; Cdk inhibitor) as previously described [11], [21]. Blebbistatin was used at 100 µM to inhibit myosin activity as previously described [45].

### Constructs

Anillin (MBP:Anillin (608–1087), MBP:Anillin (608–1087; AFAINA), MBP:Anillin (608–940) constructs were previously described [30]. In addition, MBP:Anillin (100–460, 671–772 and 772–940), GST:Anillin (608–940, 820–940 and 890–940) were generated by PCR and cloning into pMal2c(Tev) and pGEX4(Tev) vectors, respectively. Myc:Ect2 (1–883), Myc:Ect2 (1–420), Myc:Ect2 (1–227) and Myc:Ect2 (421–883) were previously described [11]. Ect2 C-terminal constructs, Ect2 (421–774, 622–883, 421–621, 622–774 and 775–883), were generated by PCR and cloned back into the c-Myc vector by PCR. Quickchange mutagenesis was used to generate the D668G mutation in Ect2. Additional mutations in Ect2 showed reduced binding to anillin similar to D668G, 667 NDC 669 – AGG, D668R and D668K. The following mutations were generated in Ect2, but showed no change in anillin binding, 627 EVD 629 – AGA, 638 SHRS 641 – AAGG, 674 RKRHK 678 – GAGHA, K708A K709A, 717 EDCHN 721 – AAAAA and 744 SDE 746 – AGA. The k-Ras Tail (EKMSKDGKKKKKKSKTKCVIM) was added to the 3′ end of Ect2 (421–883), and first cloned into pEGFP2 (BD Biosciences). The D668G mutation was introduced by quickchange mutagenesis (see above). Ect2 (421–883; Ras Tail) and Ect2 (421–883; D668G; Ras Tail) were then cloned into the cMyc vector. GFP:MyoGEF (159–531 and 352–531) constructs were generated using PCR to amplify fragments from MyoGEF cDNA (generously provided by Q. Wei, Kansas State University, Manhattan, KS) and cloned into pEGFP-C1. The GST:RBD from Rhotekin was previously described [30]. Myc:Pebble (370–853; C-term) was cloned into the c-Myc vector using PCR from Pebble cDNA (generously provided by G. Hickson, University of Montreal, Montreal, QC), and GST: Dm anillin (800–1100; AHD) was cloned into pGEX4(Tev) by PCR from *Drosophila* anillin cDNA (generously provided by G. Hickson, University of Montreal, Montreal, QC).

### Pulldowns, PIP strips and *in vitro* Binding

Transfected Hela or HEK-293 cells were lysed in 50 mM Tris pH7.6, 150 mM NaCl, 5 mM MgCl_2_, 0.5% Triton X-100, 1 mM DTT with protease inhibitors (1 mM PMSF, 10 mg/mL each leupeptin and pepstatin) and incubated with 5 µg of purified MBP or GST-tagged anillin protein at 4°C to pull down Ect2 (final concentrations ranged from 200–500 nM). Proteins were bacterially expressed and purified as previously described [30]. MBP fusions were purified using amylose resin (NEB) and GST fusions were purified using glutathione sepharose (GE) in the same buffer described above. All proteins were quantitated by coomassie-stained gels of aliquots run by SDS-PAGE, and comparing the intensity of the protein to a known concentration of standard protein (BSA). To pull down active RhoA, GST:RBD was bacterially expressed and purified with glutathione sepharose (GE) as previously described [30], [37]. Cells at ∼90–100% confluency from one 10 cm plate were lysed rapidly on ice in 400 µL of buffer and bound to 5–10 µg freshly prepared GST:RBD beads for 90 minutes (final concentration ∼1 µM), then washed twice with 1 mL wash buffer (same as for lysis, but without protease inhibitors). PIP strips (Echelon Biosciences) were incubated with lysates from three 10 cm plates of transfected HEK-293 cells.

*In vitro* binding was performed using purified, recombinant proteins, as described above, in 150 µL buffer containing 50 mM Tris pH7.6, 150 mM NaCl, 5 mM MgCl_2_ and 0.5% Triton X-100. Soluble MBP was also included in the reaction to saturate the beads. Cleaved Ect2 fragments (two different amounts −0.5 µg and 1.5 µg) in solution were added to buffer with 5 µg of MBP or MBP:anillin (AHD), and proteins were incubated with rotating at room temperature for 1 hour, or at 4°C for 2 hours. Beads were washed 4–5× with buffer before adding SDS sample buffer to denature the proteins for SDS-PAGE.

All pull downs were run by SDS-PAGE and wet-transferred to nitrocellulose membrane (low autofluorescence) for western blotting. All blots were reversibly stained with ponceau to check for transfer efficiency. The following primary antisera were used for western blots or PIP strips, mouse anti-Myc antibodies (generously provided by Dr. Sacher, Concordia University, Montreal, QC) were used directly, 1∶1000 mouse anti-GFP antibodies (Roche), 1∶5000 rabbit anti-anillin antibodies [30], 1∶1000 rabbit anti-Ect2 antibodies [11], 1∶1000 mouse anti-Cyk-4 antibodies (Abnova) and 1∶300 mouse anti-RhoA antibodies (Santa Cruz). The following secondary antisera were used at a 1∶2500 dilution, anti-mouse 680 (Rockland), anti-rabbit 800 (Rockland) and anti-mouse Alexa 488 (Invitrogen). Depending on the fluorophore, blots were scanned at 700 and/or 800 wavelengths using the Odyssey scanner (Li-Cor Biosciences) or at 488 nm using the Typhoon Trio phosphoimager (GE). Using Image J, bands were quantitated based on regions of interest and pixel intensities on the original 16-bit images. Images were converted to 8-bit by Image J, then made into figures using Adobe Photoshop and Illustrator (Adobe).

### Immunoflourescence

Cells were fixed in 100% cold methanol or 10% cold TCA as previously described [11]. The following primary antisera were used for immunofluorescence, 1∶2 dilution of supernatant containing mouse anti-Myc antibodies (generously provided by Dr. Sacher, Concordia University, Montreal, QC), 1∶50 mouse anti-RhoA antibodies (Santa Cruz), 1∶200 mouse anti-GFP antibodies (Roche, Laval, QC), 1∶50 mouse anti-Plk1 antibodies (Santa Cruz), 1∶200 rabbit anti-anillin antibodies [30], 1∶200 rabbit anti-Ect2 antibodies [11], 1∶200 mouse anti-tubulin antibodies (DM1A, Sigma-Aldrich), 1∶50 rabbit anti-nonmuscle myosin II antibodies (Cell Signaling) and DNA was visualized with 1∶1000 1 mg/mL DAPI (Sigma-Aldrich). The following secondary antisera were used at 1∶250–1∶500 dilution, anti-mouse Alexa 488 (Invitrogen), anti-rabbit Alexa 568 (Invitrogen), anti-mouse Alexa 568 (Invitrogen) and anti-rabbit Alexa 488 (Invitrogen). Images were captured on a Leica DMI6000B microscope (Leica Microsystems) with the 40×/0.75 or 63×/1.4 NA objectives and the Hamamatsu OrcaR2 camera using Volocity acquisition software (PerkinElmer). The image in Figure 1B was acquired using a 63×/1.4 NA objective on a Zeiss Axiovert 200 M microscope equipped with a Yokogawa CSU-10 spinning-disk unit (McBain) and a 50 mW, 473 nm and 561 nm DPSS lasers (Cobolt), with a Cascade 1 kb camera (Photometrics) and Metamorph (Molecular Devices) acquisition software. Images were opened in Image J, adjusted to control levels, then converted to 8-bit images before importing them into Adobe Photoshop and Illustrator (Adobe). To visualize co-localization or perform quantitations, 0.1–0.5 µm Z stacks were acquired using the piezo Z stage (Mad City Labs) on both microscopes. Images were converted into maximum intensity Z-stack projections and cell measurements were performed in Image J on the 16-bit images, and graphs were generated using Excel (also calculations; Microsoft).

## Supporting Information

Figure S1 **The PH region of Ect2 interacts with the AHD of Anillin.** A) A western blot of lysates from HEK-293 cells transfected with Myc: Ect2 FL, E1 (N-term) and E3 (C-term) pulled down with GST or GST:AHD (A2) of anillin and stained for Myc. A ponceau stain of the blot is shown below. B) Z-stack projections of fixed Hela cells transfected with Myc:Ect2 (E3) co-stained with anillin (green), Myc (red) and DAPI (blue). Yellow boxes show zoomed in regions. Scale bar is 10 µm. C) A western blot of lysates from HEK-293 cells transfected with Myc-tagged Ect2 (E3) pulled down with MBP tagged wt or mutant (837 DFEINIE 843 - AFAINIA) anillin C-term and stained for Myc. D) Western blots of lysates from HEK-293 cells expressing either Myc-tagged Ect2 (E3) or a GFP:MyoGEF fragment the PH region (352–531) pulled down with GST tagged AHD of anillin stained for Myc or GFP. A ponceau stain of the blot is shown below. E) A western blot of lysates from HEK-293 cells transfected with Myc-tagged Ect2 (E3) pulled down with GST or GST tagged anillin fragments (A5 and A6) and stained for Myc.
(TIF)

Figure S2 **Anillin interacts with Ect2 at the cortex.** A) An amino acid sequence alignment of the PH region from human, *Drosophila* and *C. elegans* Ect2 performed using Clustal W is shown. Residues shaded red are hydrophobic, blue are acidic, purple are basic and green are neutral. Stars indicate identical residues. B) Western blots compare the difference in wt Ect2 (C-term) vs. D668G mutant binding to GST:AHD. The western blot on the left shows lysates from HEK-293 cells transfected with Myc-tagged Ect2 (C-term) diluted to 0.5, 0.25 and 0.05-fold respectively, pulled down with GST:AHD and probed for Myc. The ponceau-stained blot is shown below. The western blot on the right is similar, except HEK-293 cells were transfected with Myc-tagged Ect2 (C-term; D668G). A graph shows the % bound protein at each dilution (wt in black and D668G in grey).
(TIF)

Figure S3 **Anillin and Ect2 are required for the cortical localization of microtubules.** A) Line plots show Ect2 fluorescence (Y-axis) along the equatorial or non-equatorial axis (dotted yellow lines) of multiple cells for control or anillin-depleted cells. An example of one cell that was plotted is shown in the upper right-hand corner. B) Z-stack projections of fixed Hela cells with anillin RNAi co-stained for tubulin (red) and anillin (green). Inverted images are shown for better contrast. C) Z-stack projections of fixed Hela cells with anillin or Ect2 RNAi, and co-stained for tubulin (green) and nonmuscle myosin II (red) and DAPI (blue). A graph shows the average ratio of tubulin fluorescence at the equatorial cortex to inside the cell. Lines show standard deviation and probabilities were calculated by the students t test.
(TIF)

Figure S4 ***Drosophila*** **Pebble does not interact with Anillin.** A western blot of lysates from HEK-293 cells transfected with Myc-tagged Pbl or Ect2 C-term constructs pulled down with GST-tagged AHD (A2) from *Drosophila* (Dm) or human (Hs) anillin probed for Myc. A ponceau stain of the blot is shown below.
(TIF)
