# Supplementary figures and images for: An Anillin-Ect2 Complex Stabilizes Central Spindle Microtubules at the Cortex during Cytokinesis
Source: PLoS One. 2012 Apr 13;7(4):e34888. doi: 10.1371/journal.pone.0034888 (PMC3325936; doi:10.1371/journal.pone.0034888)

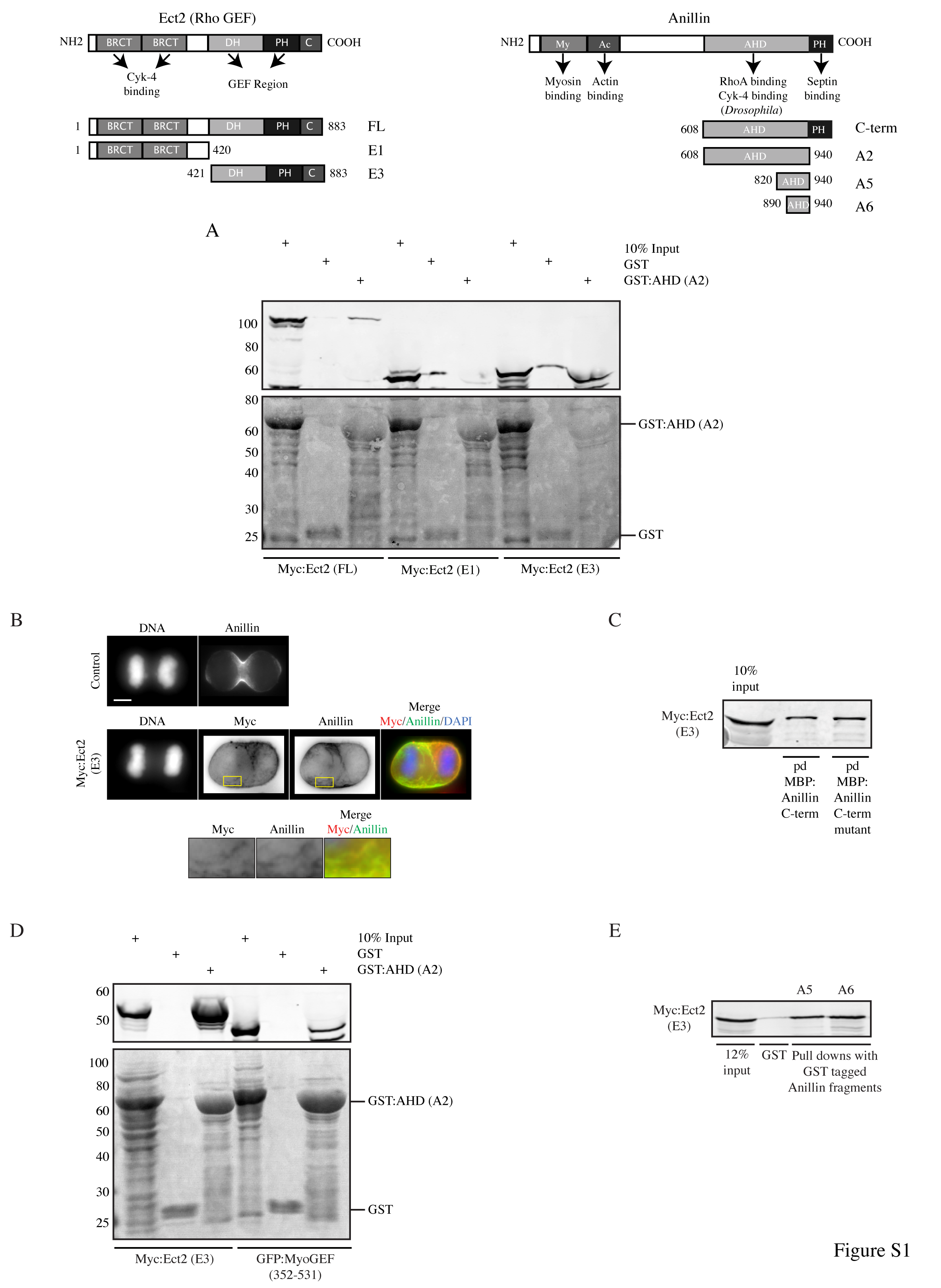

Supplement: Figure S1 — The PH region of Ect2 interacts with the AHD of Anillin. A) A western blot of lysates from HEK-293 cells transfected with Myc: Ect2 FL, E1 (N-term) and E3 (C-term) pulled down with GST or GST:AHD (A2) of anillin and stained for Myc. A ponceau stain of the blot is shown below. B) Z-stack projections of fixed Hela cells transfected with Myc:Ect2 (E3) co-stained with anillin (green), Myc (red) and DAPI (blue). Yellow boxes show zoomed in regions. Scale bar is 10 µm. C) A western blot of lysates from HEK-293 cells transfected with Myc-tagged Ect2 (E3) pulled down with MBP tagged wt or mutant (837 DFEINIE 843 - AFAINIA) anillin C-term and stained for Myc. D) Western blots of lysates from HEK-293 cells expressing either Myc-tagged Ect2 (E3) or a GFP:MyoGEF fragment the PH region (352–531) pulled down with GST tagged AHD of anillin stained for Myc or GFP. A ponceau stain of the blot is shown below. E) A western blot of lysates from HEK-293 cells transfected with Myc-tagged Ect2 (E3) pulled down with GST or GST tagged anillin fragments (A5 and A6) and stained for Myc. (TIF) [file pone.0034888.s001.tif]

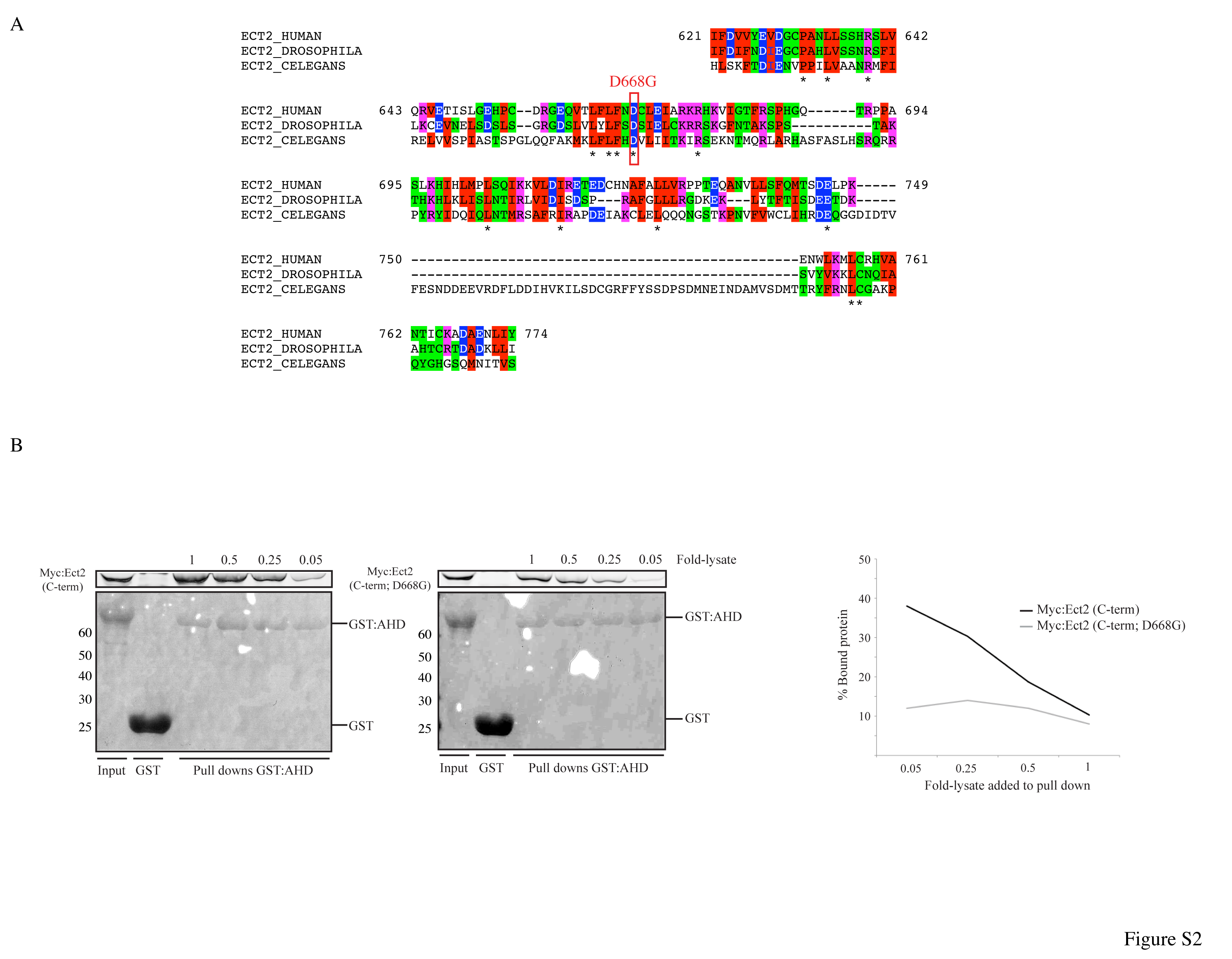

Supplement: Figure S2 — Anillin interacts with Ect2 at the cortex. A) An amino acid sequence alignment of the PH region from human, Drosophila and C. elegans Ect2 performed using Clustal W is shown. Residues shaded red are hydrophobic, blue are acidic, purple are basic and green are neutral. Stars indicate identical residues. B) Western blots compare the difference in wt Ect2 (C-term) vs. D668G mutant binding to GST:AHD. The western blot on the left shows lysates from HEK-293 cells transfected with Myc-tagged Ect2 (C-term) diluted to 0.5, 0.25 and 0.05-fold respectively, pulled down with GST:AHD and probed for Myc. The ponceau-stained blot is shown below. The western blot on the right is similar, except HEK-293 cells were transfected with Myc-tagged Ect2 (C-term; D668G). A graph shows the % bound protein at each dilution (wt in black and D668G in grey). (TIF) [file pone.0034888.s002.tif]

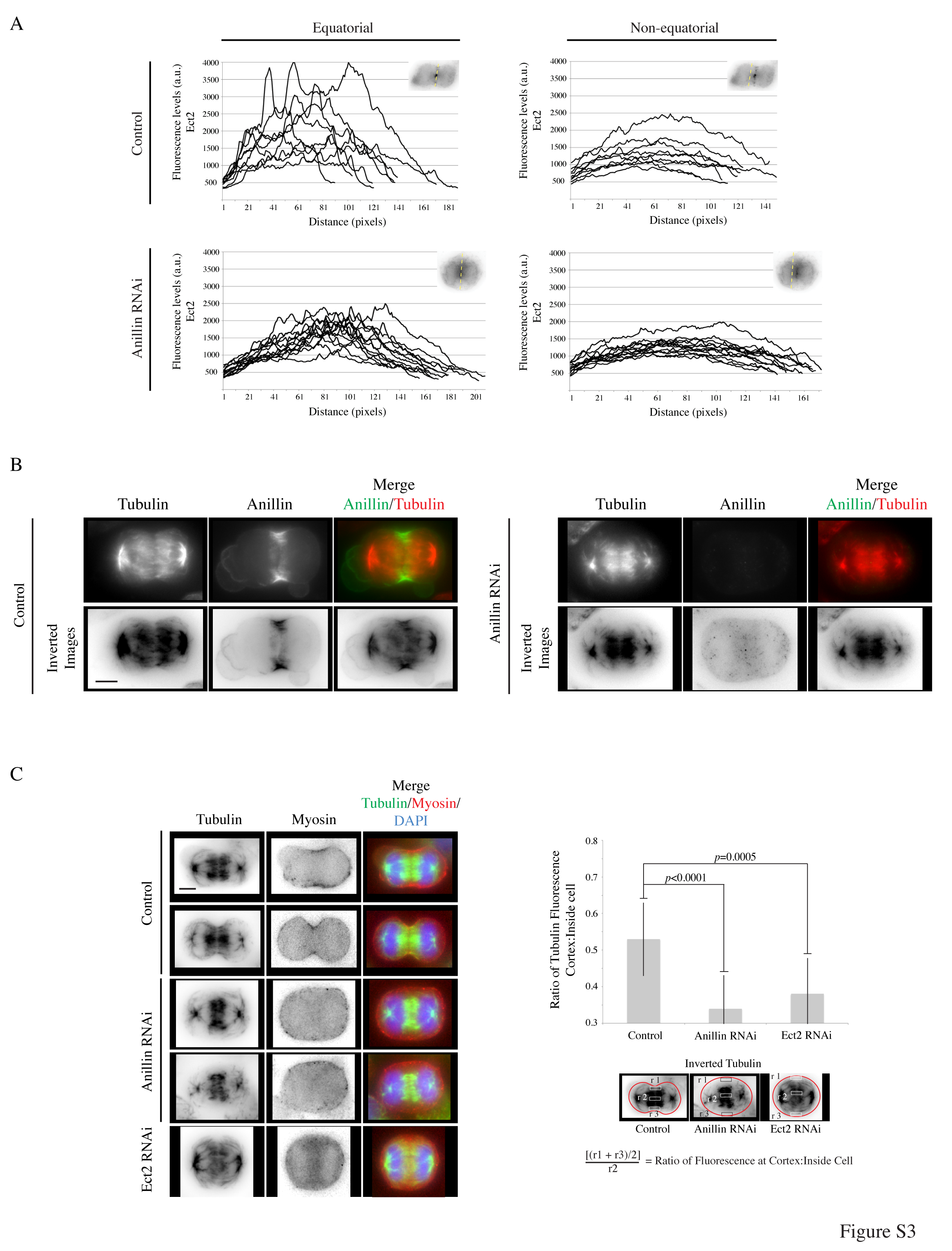

Supplement: Figure S3 — Anillin and Ect2 are required for the cortical localization of microtubules. A) Line plots show Ect2 fluorescence (Y-axis) along the equatorial or non-equatorial axis (dotted yellow lines) of multiple cells for control or anillin-depleted cells. An example of one cell that was plotted is shown in the upper right-hand corner. B) Z-stack projections of fixed Hela cells with anillin RNAi co-stained for tubulin (red) and anillin (green). Inverted images are shown for better contrast. C) Z-stack projections of fixed Hela cells with anillin or Ect2 RNAi, and co-stained for tubulin (green) and nonmuscle myosin II (red) and DAPI (blue). A graph shows the average ratio of tubulin fluorescence at the equatorial cortex to inside the cell. Lines show standard deviation and probabilities were calculated by the students t test. (TIF) [file pone.0034888.s003.tif]

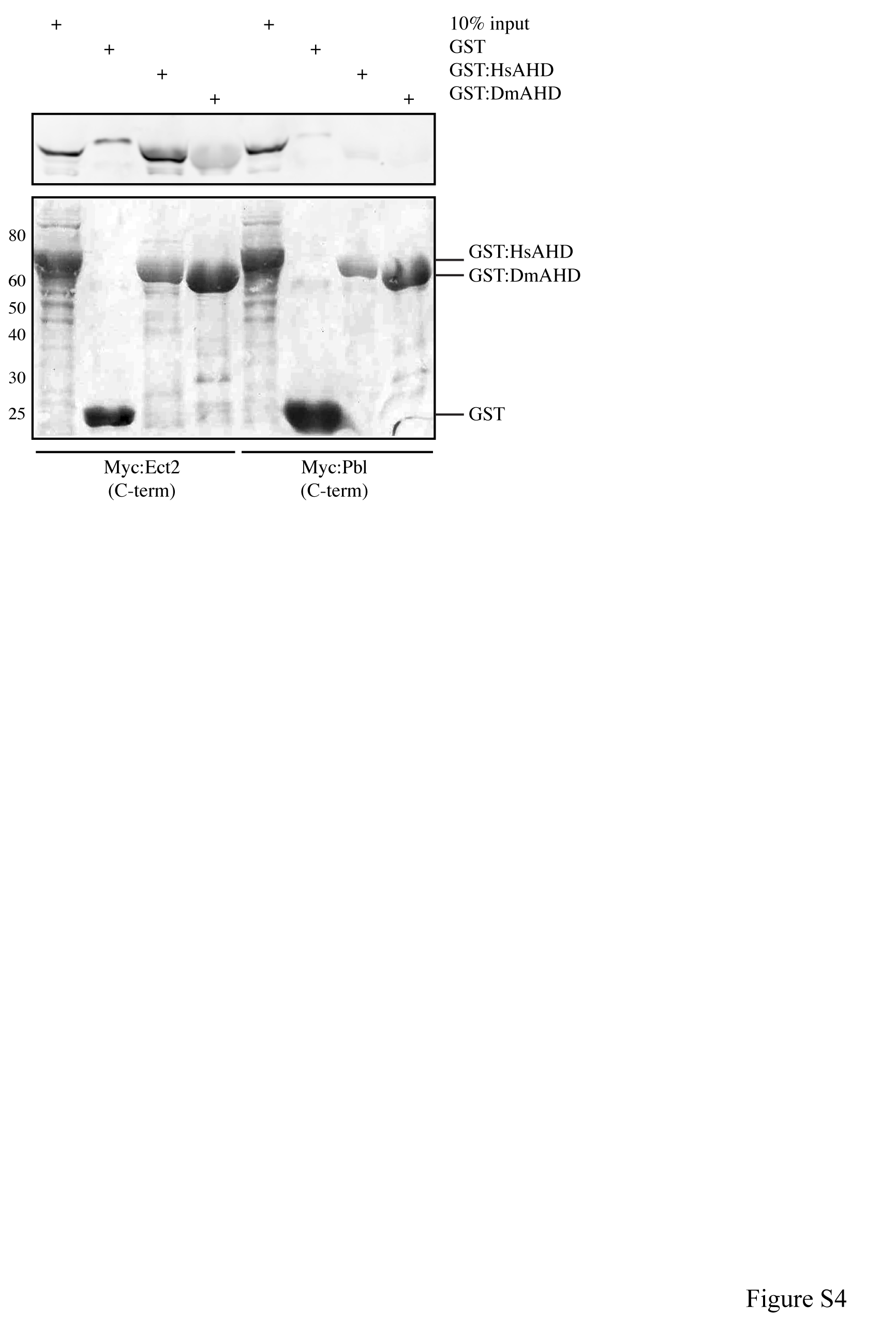

Supplement: Figure S4 — Drosophila Pebble does not interact with Anillin. A western blot of lysates from HEK-293 cells transfected with Myc-tagged Pbl or Ect2 C-term constructs pulled down with GST-tagged AHD (A2) from Drosophila (Dm) or human (Hs) anillin probed for Myc. A ponceau stain of the blot is shown below. (TIF) [file pone.0034888.s004.tif]
